# Phenotypic Spectrum and Limb-Length Discrepancy in Congenital Lower Limb Deficiencies: A Tertiary Care Center Experience

**DOI:** 10.3390/children13081042

**Published:** 2026-08-05

**Authors:** Fahad Alshayhan, Mishari Alanezi, Abdullah Addar, Abdulaziz S. AlNahari, Fahad Alhuzaimi, Waleed Albishi

**Affiliations:** 1 Department of Orthopedic Surgery, College of Medicine, King Saud University, Riyadh 11362, Saudi Arabia; 2 College of Medicine, King Saud University, Riyadh 11362, Saudi Arabia

**Keywords:** lower-limb deficiency, hemimelia, limb-length discrepancy, pediatrics, deformities

## Abstract

**Highlights:**

**What are the main findings?**

- Proximal femoral focal deficiency was the most common congenital lower-limb deficiency in this tertiary cohort and was predominantly represented by milder, unilateral phenotypes.
- Combined proximal femoral focal deficiency and fibular hemimelia exhibited the greatest limb-length discrepancy and a higher burden of associated anomalies compared with isolated deficiencies.

**What are the implications of the main findings?**

- Standardized phenotypic classification and comprehensive whole-limb assessment are essential for guiding reconstructive planning and surveillance in congenital lower-limb deficiencies.
- This study provides one of the few structured regional cohorts and supports the development of multicenter registries and future outcome-based research in rare congenital limb deficiencies.

**Abstract:**

Background/Objectives: Congenital lower-limb deficiencies, including proximal femoral focal deficiency (PFFD), fibular hemimelia (FH), and tibial hemimelia (TH), are rare, phenotypically heterogeneous disorders with important implications for reconstructive planning. However, phenotypic data remain limited. This study aimed to characterize the clinical and radiological spectrum of these deficiencies and evaluate associated anomalies and limb-length discrepancy (LLD) across diagnostic groups. Materials and Methods: A retrospective cohort study included all patients diagnosed with PFFD, FH, or TH at a tertiary referral center between January 2021 and December 2025. Demographic, clinical, and radiographic data were extracted from electronic medical records. Patients were classified using established systems, and associated anomalies, distal morphology, laterality, and LLD were evaluated. Descriptive statistics and exploratory non-parametric comparisons across diagnostic groups were performed. Results: A total of 143 patients were included: 91 (63.6%) with PFFD, 23 (16.1%) with FH, 10 (7.0%) with TH, 18 (12.6%) with combined PFFD + FH, and 1 (0.7%) with combined PFFD + TH. Isolated PFFD was predominantly unilateral and clustered within the milder spectrum, most commonly Paley type 1a and Aitken type A. In contrast, FH was dominated by the severe Paley type 3b phenotype, whereas TH showed marked classification heterogeneity. Upper-limb anomalies were more common in FH and TH than in isolated PFFD. LLD differed significantly among diagnostic groups (*p* = 0.003), with the greatest median discrepancies observed in combined PFFD + FH and isolated PFFD. Conclusions: Congenital lower-limb deficiencies are highly heterogeneous, with combined deficiencies exhibiting greater LLD and anomaly burden. These findings provide structured regional data to support classification and reconstructive planning.

## 1. Introduction

Congenital lower-limb deficiencies represent a heterogeneous group of rare developmental anomalies characterized by partial or complete absence of one or more skeletal segments of the lower extremity. Such anomalies arise from disruptions in limb bud development during early embryogenesis and may result in substantial anatomical, functional, and psychosocial challenges throughout life [1]. Although individually uncommon, they carry significant clinical implications, as they may affect limb length, alignment, joint stability, gait mechanics, and overall functional mobility from an early age [2]. Among the spectrum of congenital longitudinal lower-limb deficiencies, proximal femoral focal deficiency (PFFD), fibular hemimelia (FH), and tibial hemimelia (TH) represent three of the principal patterns encountered in pediatric orthopedic practice [2,3]. PFFD is characterized by varying degrees of femoral hypoplasia associated with abnormalities of the proximal femur and hip joint morphology. The clinical presentation ranges from isolated femoral shortening with relatively preserved hip anatomy to severe proximal femoral deficiency accompanied by substantial disruption of hip stability and joint congruity. The reported incidence of PFFD is estimated to range between 1.1 and 2.0 per 100,000 live births [3]. FH is considered the most common congenital long-bone deficiency of the lower extremity, with reported incidence estimates ranging from 7.4 to 20 cases per million live births [4]. It includes a broad phenotypic spectrum extending beyond fibular absence to include tibial shortening and bowing, ankle and hindfoot deformities, genu valgum, knee instability, and femoral shortening [5,6]. TH, in contrast, is an extremely rare anomaly with an estimated incidence of approximately 1 per million live births [7]. It represents a preaxial longitudinal deficiency characterized by partial or complete tibial absence, frequently accompanied by abnormal knee and ankle morphology [8]. Accurate phenotypic characterization of these congenital limb deficiencies is essential, as anatomical variation directly influences prognosis, surveillance strategies, and the feasibility of limb reconstruction [2,8,9]. Despite the clinical importance of these entities, the existing literature remains relatively limited and is largely derived from institutional series reported in North America and Europe. Furthermore, many studies focus primarily on surgical techniques or individual case reports rather than providing comprehensive epidemiologic analyses of affected populations. Region-specific data from the Middle East and North Africa (MENA) remain particularly limited. In Saudi Arabia, congenital anomalies are recognized as an important health burden, although current data are largely confined to isolated case reports rather than dedicated cohort studies [10,11]. Therefore, this study aimed to address this gap by presenting the clinical and radiological characteristics of patients with PFFD, FH, and TH managed at our high-volume tertiary care referral center, and to develop a regional database for these rare congenital lower-limb deficiencies.

## 2. Materials and Methods

### 2.1. Study Design, Settings, and Population

This retrospective cohort study was conducted at King Saud University Medical City, a tertiary-care and referral center in Riyadh, Saudi Arabia. The institution functions as a high-volume center that receives referrals across the country for managing complex pediatric deformities. All patients evaluated in the pediatric orthopedic clinics between January 2021 and December 2025 were screened for eligibility. Patients were included if they had a confirmed diagnosis of PFFD, FH, or TH based on clinical assessment and radiological evaluation, and if complete clinical and radiological records were available for review. While patients with incomplete medical records or missing essential imaging variables to an extent that precluded accurate classification were excluded. The study was approved by the Institutional Review Board (IRB) of the College of Medicine, King Saud University (Project No. E-26-5249). Given the retrospective nature of the study, the IRB committee waived the requirement for informed consent. All data were handled in a de-identified manner prior to analysis, and patient confidentiality was maintained throughout the study. Additionally, the manuscript was prepared in accordance with the Strengthening the Reporting of Observational Studies in Epidemiology (STROBE) statement for observational studies [12].

### 2.2. Data Collection and Variables

Data were extracted from Electronic Medical Records (EMRs) and recorded in a computerized database. Collected variables included demographic characteristics (age at presentation and sex) and clinical characteristics such as laterality, the presence of associated upper-limb anomalies, and the type of limb deficiency (PFFD, FH, or TH). Radiological variables were obtained through review of plain radiographs. Radiographic assessments were performed by a fellowship-trained pediatric orthopedic surgeon specialized in limb deformity correction. Radiographic variables included classification categories and condition-specific anatomical features relevant to each deficiency pattern. For PFFD, radiological variables included the Paley and Aitken classifications and femoral alignment. For FH, variables included the Paley and Achterman–Kalamchi classifications, number of foot rays, presence of a ball-and-socket ankle joint, and ankle alignment. For TH, variables included the Paley, Jones, and Weber classifications and ankle alignment. Additionally, LLD was measured radiographically using centograms. Limb length was determined as the distance from the center of the femoral head proximally to the center of the tibial plafond distally, and LLD was calculated as the absolute difference between the affected and contralateral limbs. Patient demographic and clinical variables were analyzed at the patient level. In contrast, radiographic classification variables were analyzed at the level of the affected limb, allowing each affected limb to be classified independently using the corresponding condition-specific classification system.

### 2.3. Statistical Analysis

Descriptive analyses were performed according to the appropriate unit of analysis. Patient-level analyses included demographic characteristics and clinical features, whereas radiographic classification analyses were performed at the affected-limb level. Normality of continuous variables was assessed using the Shapiro–Wilk test in conjunction with visual inspection of histograms and Q–Q plots. Continuous variables were summarized as median with interquartile range (IQR) due to non-normal distributions, while categorical variables were presented as frequencies and percentages. Exploratory association analyses examined differences in LLD according to gender, laterality, and diagnostic group, using the Mann–Whitney U test for two-group comparisons and the Kruskal–Wallis test for comparisons across more than two groups. A two-tailed *p*-value < 0.05 was considered statistically significant. All statistical analyses were performed using IBM SPSS Statistics version 26.0 (IBM Corporation, Armonk, NY, USA).

## 3. Results

### 3.1. Demographic and Clinical Characteristics

A total of 143 patients were included: 91 (63.64%) with PFFD, 23 (16.08%) with FH, 10 (6.99%) with TH, 18 (12.59%) with combined PFFD + FH, and 1 (0.70%) with combined PFFD + TH. The demographic and clinical characteristics are summarized in Table 1. Regarding sex distribution, the isolated PFFD and FH groups were broadly balanced, with males accounting for 50.55% and 47.83%, respectively. In contrast, TH showed a male predominance (70%), whereas females were more frequent in the combined PFFD + FH group (61.11%) (Table 1). In terms of laterality, most cases were unilateral. Unilateral involvement occurred in 93.41% of PFFD, 73.91% of FH, and 60% of TH cases. Similarly, all patients with combined PFFD + FH had unilateral involvement. Additionally, associated upper-limb anomalies were uncommon in isolated PFFD (3.30%) and combined PFFD + FH (5.56%) but were more frequent in FH (21.74%) and TH (30%). The single patient with combined PFFD + TH presented at 11 months of age, was male, had unilateral involvement, an LLD of 17.1 cm, and an associated radial club hand deformity.

### 3.2. PFFD Phenotype and Classification

Among affected PFFD limbs, Paley type 1a was the predominant phenotype, accounting for 68 (74.73%), followed by type 1c and type 2b, each observed in 7 (7.69%). The remaining Paley subtypes were infrequent, and no limbs were classified as types 3b, 3c, or 4. Within the subgroup of Paley type 1a limbs, a femoral alignment flag was recorded as present in 28 (41.18%). Using the Aitken classification, type A was the most frequent category, reported in 69 (75.82%), followed by type B in 13 (14.29%), type D in 12 (13.19%), and type C in 2 (2.20%) (Table 2).

### 3.3. FH Phenotype and Radiological Characteristics

Among affected FH limbs, Paley type 3b was the most common phenotype, observed in 15 limbs (65.22%), followed by type 2 in 6 limbs (26.09%) and type 1 in 5 limbs (21.74%). According to the Kalamchi classification, type 1a predominated (13 limbs, 56.52%), while type 2 and type 1b were observed in 10 (43.48%) and 4 limbs (17.39%), respectively. The number of foot rays differed by side, with a median of 4.00 (IQR 4.00–5.00) on the right and 5.00 (IQR 5.00–5.00) on the left. A ball-and-socket ankle was observed in 5 limbs (21.74%), whereas 18 (78.26%) had no such configuration. Most limbs demonstrated normal ankle alignment (78.26%) (Table 3).

### 3.4. TH Phenotype and Classification

Among affected TH limbs, the Paley classification showed a heterogeneous phenotypic distribution. Types 3a and 5a were the most frequent categories, each observed in 4 limbs (40%), followed by type 1 in 2 limbs (20%) (Table 4). Using the Jones classification, type IV predominated, occurring in 9 (90%), while type Ia was identified in 5 (50%). According to the Weber classification, types II and VIIb were the most frequent categories, each reported in 5 (50%), followed by type I in 2 (20%). Types IIIa and Va were each observed in 1 (10%) limb, while the remaining Weber categories were not represented. Ankle alignment was evenly distributed between normal and equinovarus alignment, each seen in 4 (40%), while equinovalgus alignment was present in 2 (20%).

### 3.5. Combined PFFD + FH Radiological Phenotype and Classification

Among patients with combined PFFD + FH, the PFFD component was predominantly Paley type 1a, observed in 14 (77.78%), followed by type 3a in 2 (11.11%); types 2a and 2b were each identified in 1 (5.56%) patient, while no cases were classified as types 1b, 1c, 2c, 3b, 3c, or 4 (Table 5). Using the Aitken classification, type A was the most frequent category (n = 11, 61.11%), followed by type B (n = 4, 22.22%), type C (n = 2, 11.11%), and type D (n = 1, 5.56%). Ankle alignment was most commonly normal (n = 13, 72.22%), while equinovalgus and equinovarus alignment were observed in 4 (22.22%) and 1 (5.56%), respectively.

For the FH component, Paley type 3b predominated, occurring in 14 (77.78%), whereas type 2 was identified in 4 (22.22%); no limbs were classified as types 1, 3a, 3c, 3d, or 4. According to the Kalamchi classification, type 2 was the most frequent category (n = 11, 61.11%), followed by type 1a (n = 6, 33.33%) and type 1b (n = 1, 5.56%). The number of foot rays was generally preserved on both sides, with a median of 5.00 for the right and left feet.

### 3.6. Limb Length Discrepancy

**Figure 1:**
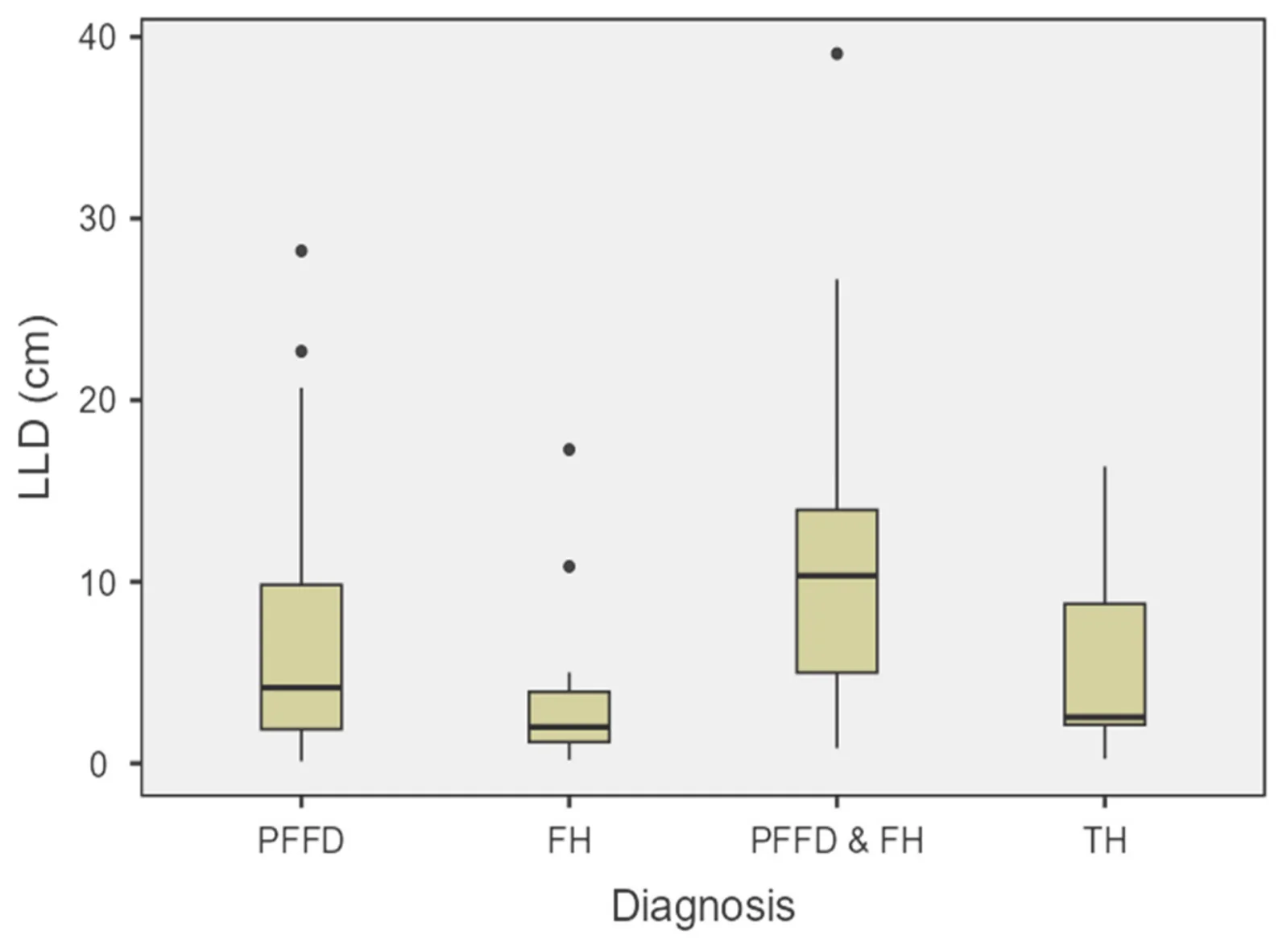
Limb length discrepancy by diagnostic group; Box-and-whisker plots showing limb-length discrepancy (LLD, cm) across the four diagnostic groups: proximal femoral focal deficiency (PFFD, *n* = 91), fibular hemimelia (FH, *n* = 23), tibial hemimelia (TH, *n* = 10), and combined PFFD + FH (*n* = 18). Boxes represent the interquartile range (IQR), the horizontal line indicates the median, whiskers extend to 1.5 × IQR, and individual points represent outlying observations.

LLD did not differ significantly by sex, with median values of 3.31 cm (IQR 1.50–7.81) in males and 4.49 cm (IQR 1.82–10.92) in females (*p* = 0.117). In contrast, laterality was significantly associated with LLD (*p* < 0.001). The highest median discrepancy was observed in right-sided cases (5.50 cm, IQR 2.42–11.08), followed by left-sided cases (4.39 cm, IQR 1.50–9.83). LLD also differed significantly across diagnostic categories (*p* = 0.003). The largest median discrepancy was observed in patients with combined PFFD + FH (10.34 cm, IQR 5.00–13.96), followed by isolated PFFD (4.17 cm, IQR 1.88–9.83). Lower values were recorded in TH (2.55 cm, IQR 2.13–8.78) and FH (2.00 cm, IQR 1.75–3.94) (Table 6, Figure 1).

## 4. Discussion

This tertiary care referral center cohort provides structured classification-based characterization of congenital longitudinal lower-limb deficiencies from a region where phenotype-level data remain limited. PFFD was the most frequent diagnosis in our series, followed by FH, combined PFFD + FH, and TH. Several clinically relevant patterns emerged. Isolated PFFD was predominantly unilateral and largely represented milder reconstructable phenotypes, whereas FH demonstrated distal-spectrum involvement affecting rays and ankle morphology. TH showed substantial heterogeneity across all applied classification systems, reflecting its recognized developmental variability. Importantly, combined PFFD + FH appeared as a reproducible clinicoradiological subgroup rather than a coincidental association. Limb-length discrepancy was greatest in combined PFFD + FH and isolated PFFD, while associated upper-limb anomalies were more frequent in FH and TH than in isolated PFFD. Together, these observations support the interpretation of congenital longitudinal lower-limb deficiencies as spectrum disorders whose radiographic expression carries direct implications for classification-based surveillance and reconstructive planning. In contrast to population-based epidemiologic reports that identify FH as the most common congenital long-bone deficiency, the predominance of PFFD in this cohort likely reflects tertiary referral enrichment of patients with proximal femoral instability and larger projected limb-length discrepancy. Accordingly, the present distribution should be interpreted as characteristic of a specialized deformity-center population rather than background incidence.

Within the PFFD subgroup, the predominance of Paley type 1a and Aitken type A indicates that most limbs in this cohort fell within the milder end of the reconstructable spectrum, with relatively preserved proximal femoral anatomy and favorable joint morphology. This distribution is clinically relevant because radiographic severity in PFFD directly influences treatment pathway selection, including suitability for limb reconstruction, staged lengthening strategies, or prosthetic-based management. Previous studies have consistently emphasized that PFFD represents a spectrum ranging from isolated congenital femoral shortening to complex proximal deficiency involving the hip, knee, and leg segments. Earlier studies have also shown that radiographic severity may not be fully stable in early childhood. Sanpera and Sparks reported that classification may evolve with growth [13], whereas Hillmann et al. highlighted the value of objective radiographic indices and delayed diagnostic confirmation as ossification progresses [14]. Maldjian et al. further demonstrated that MRI can refine classification before complete ossification of cartilaginous structures and may correct the tendency of plain radiographs to overestimate the degree of deficiency [15]. These observations are relevant because they indicate that phenotype-based categorization in PFFD is not only descriptive, but also closely linked to prosthetic fitting, gait strategy, and counselling regarding reconstructive versus prosthetic pathways in severe disease. In this context, the predominance of milder PFFD categories in the present cohort may reflect both the underlying phenotype distribution and a follow-up pattern in which more clearly classifiable, reconstructable cases are more likely to remain under long-term tertiary care.

Our findings in the FH subgroup are consistent with the current understanding of congenital fibular deficiency as a whole-limb disorder rather than a deformity confined to the fibula alone. In the present cohort, Paley type 3b was the most frequent phenotype, most ankles demonstrated preserved alignment, a ball-and-socket ankle configuration was observed in approximately one-fifth of cases, and the number of foot rays was often maintained. This distribution reflects the recognized distal phenotypic variability of FH and supports the interpretation of fibular deficiency as a spectrum condition involving multiple segments of the lower limb rather than an isolated longitudinal absence. The original series by Achterman and Kalamchi demonstrated that congenital fibular deficiency is commonly associated with abnormalities extending beyond the fibula itself, including femoral shortening and knee instability [16]. Subsequently, Birch et al. and Hamdy et al. further characterized FH as a spectrum disorder encompassing tibial deformity, ray deficiency, ankle and hindfoot malalignment, and variable knee involvement [6,17]. Fuller et al. and Paley similarly emphasized that contemporary evaluation of FH should incorporate projected limb-length discrepancy and foot morphology rather than relying solely on the degree of fibular absence [5,18]. Searle et al. further demonstrated that even patients with radiographically normal fibulae may still fall within the FH spectrum when associated features such as absent lateral rays, ball-and-socket ankle, tarsal coalition, and limb shortening are present [19]. In their mixed congenital limb-deficiency cohort, Kothari et al. demonstrated that FH was associated with substantial distal phenotypic heterogeneity, with equinocavovarus deformity observed in 24% of feet, equinovalgus in 71%, and ray deficiency occurring predominantly in an intermediate rather than purely lateral distribution. Importantly, the presence of tarsal coalition was not associated with ball-and-socket ankle morphology [20]. These observations help explain why preservation of five rays and apparently modest ankle malalignment in some limbs should not be interpreted as excluding FH, but rather as part of its recognized distal phenotypic range. More recently, Couvreur et al. reported frequent morphological abnormalities and substantial predicted LLD in a multicenter French cohort [21]. In this context, our findings support the interpretation that the distal phenotype of FH is variable but still follows recognizable patterns, and that preservation of five-foot rays in a considerable proportion of limbs does not exclude the diagnosis but rather reflects the known breadth of this disorder.

The TH subgroup demonstrates why multiple classification systems remain necessary for accurate characterization of this rare and phenotypically heterogeneous deficiency. In our cohort, substantial variability was observed across the Paley, Jones, and Weber systems, with representation of both relatively limited and clearly severe forms, together with variable ankle alignment. This distribution reflects the recognized morphological breadth of TH and underscores the limitations of relying on a single radiographic framework for clinical description and treatment planning. Jones et al. originally established the classic radiographic classification for tibial deficiency; however, subsequent studies have highlighted important limitations in its ability to capture the full spectrum of anatomical variation [22]. Weber’s classification addressed some of these gaps by incorporating the cartilaginous anlage and whole-leg morphology, thereby improving both descriptive completeness and therapeutic relevance [23]. Consistent with this, Clinton and Birch, in their 37-year institutional experience, reported that a meaningful proportion of limbs could not be classified using the Jones system alone and also demonstrated a high prevalence of associated anomalies [24]. Similarly, Litrenta et al. and Paley emphasized the marked phenotypic variability of TH, including differences in knee function, patellar development, fibular morphology, foot deformity, and associated abnormalities of the hand and femur [7,8]. Distal morphology represents another important source of heterogeneity in TH. Kothari et al. reported equinocavovarus posture in all TH feet within their cohort, with most preserved limbs requiring corrective foot and ankle procedures [20]. In comparison, the more variable ankle-alignment pattern observed in our series likely reflects both the intrinsic spectrum of distal involvement in TH and the limited subgroup size. Accordingly, these findings should be interpreted cautiously but remain consistent with the recognized clinicoradiological diversity of tibial hemimelia.

The combined PFFD + FH subgroup represents one of the most distinctive features of the present series and warrants particular attention. In these patients, the femoral component was generally mild to moderate according to PFFD classification, whereas the fibular component was most often classified as Paley 3b/Kalamchi 2, with predominantly preserved five-ray feet. This distribution is clinically relevant because the coexistence of femoral and fibular deficiency has long been considered more than a coincidental association and may reflect a shared developmental disturbance. Sorge et al. proposed that combined PFFD and fibular hemimelia may reflect a developmental field defect [25], and Walker et al. later demonstrated that children with femoral and/or fibular deficiency carry a meaningful burden of upper-extremity anomalies, particularly when the lower-limb deficiency is bilateral [26]. Similarly, Achterman and Kalamchi documented frequent femoral abnormalities among patients with congenital fibular deficiency [16]. More recently, Chomiak et al. showed that foot and ankle abnormalities form an important part of the PFFD phenotype, while the degree of ray deficiency and coalition appears to vary according to associated fibular involvement rather than femoral severity alone [27]. Taken together, these observations support the interpretation that the combined PFFD + FH subgroup in our cohort represents a recognizable developmental pattern with a characteristic distal morphological profile rather than a simple overlap between two otherwise independent deficiencies, reinforcing the importance of whole-limb assessment when counselling patients and planning reconstructive strategy.

Isolated PFFD in our cohort was predominantly unilateral, with near-equal left–right distribution and relatively limited bilateral involvement, consistent with the established description of PFFD as primarily a unilateral deficiency [3]. In contrast, bilateral involvement was more frequent in FH and particularly in TH. Although the proportion of bilateral TH cases in our series appears higher than the approximately one-third reported in previous reviews, this difference is plausible given the small subgroup size and the referral patterns typical of tertiary deformity centers.

The distribution of associated upper-limb anomalies provides additional insight into the developmental context of these deficiencies. Such anomalies were uncommon in isolated PFFD but occurred more frequently in FH and TH, and the single patient with combined PFFD + TH presented with a radial club hand deformity. Walker et al. reported upper-extremity anomalies in 56 of 327 children with femoral and/or fibular deficiency and demonstrated a higher prevalence among patients with bilateral lower-limb involvement [26]. Similarly, Clinton and Birch documented associated anomalies in 79% of patients with tibial deficiency [24], while Litrenta et al. highlighted abnormalities of the hand and femur as common orthopedic associations in TH [8]. These comparisons suggest that the associated-anomaly burden observed in our FH and TH groups is not incidental and supports a low threshold for comprehensive musculoskeletal screening, particularly in bilateral and combined presentations.

LLD represents one of the most clinically relevant findings of the present study and serves as an important marker of phenotypic severity across congenital longitudinal lower-limb deficiencies. In our cohort, discrepancy did not differ significantly by sex but varied according to laterality and diagnostic group, with the largest median values observed in combined PFFD and FH, followed by isolated PFFD, whereas FH and TH demonstrated lower median discrepancies overall. This distribution is consistent with the established role of femoral deficiency as a principal contributor to projected limb shortening. In PFFD, shortening is central to the natural history of the disorder and remains a key determinant of reconstructive planning [2]. In FH, both Birch’s classification and subsequent reviews by Hamdy and Fuller emphasize predicted limb shortening alongside foot functionality as primary factors guiding treatment selection [5,6,17]. In a French multicenter FH cohort, the predicted mean discrepancy reached 10.3 cm and the median final discrepancy was reduced to less than 2 cm after management; however, 37% of patients still lacked plantigrade support and lengthening procedures were associated with a 73% complication rate, indicating that discrepancy correction alone does not fully address the functional burden of the disorder [21]. Similarly, Mishima et al., in a lengthening series of eight patients, achieved correction to within 11 mm but reported regenerate fractures related to insufficient callus maturation and persistent lateral mechanical-axis deviation as ongoing challenges [28]. Together, these findings support the interpretation that limb-length discrepancy functions not only as a marker of anatomical severity but also as a central determinant of treatment strategy, and should therefore be considered alongside alignment, foot morphology, and complication risk when planning reconstructive pathways in congenital lower-limb deficiency.

Although treatment outcomes were beyond the scope of this study, the phenotypic patterns identified may have important clinical implications. Standardized characterization of congenital lower-limb deficiencies can facilitate multidisciplinary evaluation, support reconstructive planning and surveillance, and improve patient counseling by providing a more consistent framework for describing disease severity. Furthermore, these findings establish a regional dataset that may serve as a foundation for future studies examining the relationship between phenotype, treatment strategy, and long-term functional outcomes.

The present study captured a 5-year consecutive institutional experience, applied predefined eligibility criteria, and analyzed a clinically relevant dataset using multiple established classification systems across PFFD, FH, TH, and combined phenotypes. Radiological assessment was performed by a fellowship-trained pediatric orthopedic surgeon specialized in limb deformity correction, and the analysis extended beyond diagnostic distribution to include laterality, associated anomalies, distal morphology, and limb-length discrepancy. Together, these features enhance the descriptive value of the dataset and make it more informative than isolated case reports or narrowly treatment-focused series. An additional strength of the study is its regional relevance. In Saudi Arabia, congenital anomalies are not uncommon, with hospital-based studies reporting overall prevalence estimates ranging from 23.0 to 41.2 per 1000 live births [29,30]. Within these data, musculoskeletal and limb malformations account for approximately 4.1 per 1000 live births, placing them among the more frequent non-cardiac anomaly groups in the Kingdom [31]. However, these figures remain largely aggregated at the organ-system level and therefore provide limited insight into rare longitudinal lower-limb deficiencies such as PFFD, FH, and TH, whose management depends on detailed phenotype characterization, joint morphology, associated anomalies, and projected limb-length discrepancy. Accordingly, the present study extends beyond case reporting by providing structured phenotypic data across multiple deficiency patterns within a tertiary referral setting. This contribution is relevant not only for regional epidemiology but also for clinical pathway development, as institutional datasets in rare limb deficiencies often represent the first practical foundation for future registries and multicenter collaboration [10,11]. Despite these strengths, several limitations should be considered when interpreting the present findings. First, the retrospective single-center design introduces referral bias and may limit generalizability beyond tertiary deformity-center populations. Second, several subgroup analyses were constrained by small sample sizes, particularly within the TH subgroup and the more severe PFFD categories, which limits interpretation of category-specific medians and precludes robust inferential comparisons. Additionally, the small number of observations in the TH subgroup and the more severe PFFD and FH classification categories warrants cautious interpretation, and these findings should not be over-interpreted until validated in larger cohorts. Third, comparisons of absolute LLD between diagnostic groups should be interpreted with caution because the median age differed across groups. As LLD generally progresses with skeletal growth, age may have acted as a confounding factor, potentially influencing comparisons of absolute LLD values. Fourth, all radiographic classifications were performed by a single fellowship-trained pediatric orthopedic surgeon specialized in limb deformity correction. Although this ensured consistency in image interpretation, inter-rater reliability could not be assessed. Future studies should incorporate multiple independent reviewers and formally evaluate inter-rater agreement to strengthen the reliability and reproducibility of phenotypic classification. Fifth, radiographic classification of congenital limb deficiency is inherently influenced by developmental stage, as phenotypes may evolve with growth and may be further refined with advanced imaging; this limitation partly explains the development of newer classification systems such as those proposed by Weber and Paley, particularly for TH. Finally, the study was designed as a descriptive phenotypic analysis and therefore does not evaluate treatment pathways, surgical decision-making, longitudinal growth prediction, functional outcomes, or health-related quality of life. Future prospective multicenter studies integrating standardized phenotypic classification with treatment strategies and longitudinal outcomes are needed to better define the prognostic and therapeutic implications of these deficiencies.

## 5. Conclusions

This tertiary-center cohort reinforces several key principles in congenital longitudinal lower-limb deficiency. PFFD, FH, and TH represent phenotypically heterogeneous disorders in which standardized radiographic classification remains essential for clinical characterization despite recognized limitations. Combined deficiency patterns appear to reflect clinically meaningful developmental associations rather than incidental overlap, and limb-length discrepancy, together with associated anomalies, provides useful indicators of overall phenotypic burden. These findings emphasize the importance of comprehensive whole-limb and extra-limb assessment, particularly in FH, TH, and combined deficiency patterns, and highlight the need for regionally coordinated registries capable of linking classification with treatment strategy, longitudinal growth, and functional outcomes. In a field still largely informed by reviews, historical institutional series, and isolated case reports, the present cohort contributes structured regional data that may help refine current phenotypic understanding and support future multicenter collaborative research.

## Figures and Tables

**Table 1:** Baseline characteristics by condition.

Characteristic		PFFD	FH	TH	PFFD + FH
Patients, n (%)		91 (63.64%)	23 (16.08%)	10 (6.99%)	18 (12.59%)
Age at presentation, years, median (IQR)		5.0 (3.00–9.00)	3.0 (1.92–7.00)	2.0 (0.42–3.52)	5.0 (2.00–6.8)
Gender, n (%)	Male	46 (50.55%)	11 (47.83%)	7 (70.00%)	7 (38.89%)
	Female	45 (49.45%)	12 (52.17%)	3 (30.00%)	11 (61.11%)
Upper limb anomalies present, n (%)		3 (3.30%)	5 (21.74%)	3 (30.00%)	1 (5.56%)

FH, fibular hemimelia; IQR, interquartile range; PFFD, proximal femoral focal deficiency; TH, tibial hemimelia.

**Table 2:** PFFD radiological phenotype and classification *.

Variable	Category	n (%)
Paley classification	Type 1a	68 (74.73%)
	Type 1b	3 (3.30%)
	Type 1c	7 (7.69%)
	Type 2a	4 (4.40%)
	Type 2b	7 (7.69%)
	Type 2c	4 (4.40%)
	Type 3a	3 (3.30%)
	Type 3b	0 (0.0%)
	Type 3c	0 (0.0%)
	Type 4	0 (0.0%)
Aitken classification	Type A	69 (75.82%)
	Type B	13 (14.29%)
	Type C	2 (2.20%)
	Type D	12 (13.19%)
Femur alignment flag **	Yes	28 (41.18%)
	No	40 (58.82%)

PFFD, proximal femoral focal deficiency. * Radiographic classifications were analyzed at the level of affected limbs rather than individual patients. Therefore, patients with bilateral involvement contributed two limbs to the analysis. ** Femoral alignment flag: Indicates the presence or absence of angular deformity or malalignment of the femur identified on radiographic assessment; the percentage was calculated out of the 68 patients with 1a Paley classification.

**Table 3:** FH phenotype and foot ankle morphology *.

Variable		Category	n (%)
Paley classification		Type 1	5 (21.74%)
		Type 2	6 (26.09%)
		Type 3a	0 (0.0%)
		Type 3b	15 (65.22%)
		Type 3c	0 (0.0%)
		Type 3d	0 (0.0%)
		Type 4	1 (4.35%)
Kalamchi classification		Type 1a	13 (56.52%)
		Type 1b	4 (17.39%)
		Type 2	10 (43.48%)
Number of rays	Right	Median (IQR)	4.00 (4.00–5.00)
	Left	Median (IQR)	5.00 (5.00–5.00)
Ball and socket ankle		Yes	5 (21.74%)
		No	18 (78.26%)
Ankle alignment		Normal	18 (78.26%)
		Equinovarus	2 (8.70%)
		Equinovalgus	3 (13.04%)

FH, fibular hemimelia; IQR, interquartile range; SD, standard deviation. * Radiographic classifications were analyzed at the level of affected limbs rather than individual patients. Therefore, patients with bilateral involvement contributed two limbs to the analysis.

**Table 4:** TH Phenotype and Classification *.

Variable	Category	n (%)
Paley Classification	Type 1	2 (20.00%)
	Type 2a	1 (10.00%)
	Type 2b	1 (10.00%)
	Type 2c	0 (0.0%)
	Type 3a	4 (40.00%)
	Type 3b	1 (10.00%)
	Type 4a	0 (0.0%)
	Type 4b	0 (0.0%)
	Type 5a	4 (40.00%)
	Type 5b	0 (0.0%)
	Type 5c	1 (10.00%)
Jones classification	Type Ia	5 (50.00%)
	Type Ib	0 (0.0%)
	Type II	0 (0.0%)
	Type III	0 (0.0%)
	Type IV	9 (90.00%)
Weber classification	Type I	2 (20.00%)
	Type II	5 (50.00%)
	Type IIIa	1 (10.00%)
	Type IIIb	0 (0.0%)
	Type IVa	0 (0.0%)
	Type IVb	0 (0.0%)
	Type Va	1 (10.00%)
	Type Vb	0 (0.0%)
	Type VIa	0 (0.0%)
	Type VIb	0 (0.0%)
	Type VIIa	0 (0.0%)
	Type VIIb	5 (50.00%)
Ankle alignment	Normal	4 (40.00%)
	Equinovarus	4 (40.00%)
	Equinovalgus	2 (20.00%)

TH, tibial hemimelia. * Radiographic classifications were analyzed at the level of affected limbs rather than individual patients. Therefore, patients with bilateral involvement contributed two limbs to the analysis.

**Table 5:** Combined PFFD and FH Radiological Phenotype and Classification *.

	Variable		Category	n (%)
PFFD	Paley classification		Type 1a	14 (77.78%)
			Type 1b	0 (0.0%)
			Type 1c	0 (0.0%)
			Type 2a	1 (5.56%)
			Type 2b	1 (5.56%)
			Type 2c	0 (0.0%)
			Type 3a	2 (11.11%)
			Type 3b	0 (0.0%)
			Type 3c	0 (0.0%)
			Type 4	0 (0.0%)
	Aitken classification		Type A	11 (61.11%)
			Type B	4 (22.22%)
			Type C	2 (11.11%)
			Type D	1 (5.56%)
	Ankle alignment		Normal	13 (72.22%)
			Equinovarus	1 (5.56%)
			Equinovalgus	4 (22.22%)
FH	Paley Classification		Type 1	0 (0.0%)
			Type 2	4 (22.22%)
			Type 3a	0 (0.0%)
			Type 3b	14 (77.78%)
			Type 3c	0 (0.0%)
			Type 3d	0 (0.0%)
			Type 4	0 (0.0%)
	Kalamchi classification		Type 1a	6 (33.33%)
			Type 1b	1 (5.56%)
			Type 2	11 (61.11%)
	Number of rays	Right	Median (IQR)	5.00 (4.25–5.00)
		Left	Median (IQR)	5.00 (4.00–5.00)
	Ball-and-Socket Ankle		Yes	0 (0.00%)
			No	18 (100.00%)

* Radiographic classifications were analyzed at the level of affected limbs rather than individual patients. Therefore, patients with bilateral involvement contributed two limbs to the analysis.

**Table 6:** Limb length discrepancy by sex, laterality, and condition.

Variables	Category	LLD (cm) Median (IQR)	*p*-Value (Exploratory)
Gender	Male	3.31 (1.50–7.81)	0.117
	Female	4.49 (1.82–10.92)	
Laterality	Right	5.50 (2.42–11.08) ^a^	<0.001
	Left	4.39 (1.50–9.83) ^a^	
Condition	PFFD	4.17 (1.88–9.83) ^a^	0.003
	FH	2.00 (1.75–3.94) ^b^	
	TH	2.55 (2.13–8.78) ^a,b^	
	PFFD + FH	10.34 (5.00–13.96) ^a^	

Data are presented as median and interquartile range. *p* values are exploratory and derived from non-parametric comparisons across groups. Superscript letters indicate post hoc groupings, where categories sharing a letter are not significantly different. FH, fibular hemimelia; IQR, interquartile range; LLD, limb length discrepancy; PFFD, proximal femoral focal deficiency; TH, tibial hemimelia.

## Data Availability

All data are available on request from the corresponding author.

## Abbreviations

The following abbreviations are used in this manuscript:

PFFD	Proximal Femoral Focal Deficiency
FH	Fibular Hemimelia
TH	Tibial Hemimelia
LLD	Limb-Length Discrepancy
MRI	Magnetic Resonance Imaging
